# miRNA-21 and miRNA-27b Expression in Saliva of Patients with Oral Lichen Planus: A Systematic Review

**DOI:** 10.3390/ijms26125824

**Published:** 2025-06-18

**Authors:** Dario Di Stasio, Fausto Fiori, Antonio Romano, Annalisa Palmieri, Laura Mosca, Juan Antonio Ruiz Roca, Pia Lopez-Jornet, Alberta Lucchese

**Affiliations:** 1 Multidisciplinary Department of Medical-Surgical and Dental Specialties, Università Degli Studi Della Campania “Luigi Vanvitelli”, 80128 Naples, Italy; dario.distasio@unicampania.it (D.D.S.); antonio.romano4@unicampania.it (A.R.); 2 Department of Medical and Surgical Sciences, University of Bologna, 40126 Bologna, Italy; annalisa.palmieri@unibo.it; 3 Department of Life Sciences, Health and Health Professions, Link Campus University, 00185 Rome, Italy; 4 Departamento de Dermatología, Estomatología, Radiología y Medicina Física University of Murcia, 30100 Murcia, Spain; jaruizroca@um.es (J.A.R.R.); majornet@um.es (P.L.-J.)

**Keywords:** oral lichen planus, miRNA, OLP, microRNA

## Abstract

Oral lichen planus (OLP) is a chronic inflammatory disorder of the oral mucosa with a recognized risk of malignant transformation. MicroRNAs, particularly miRNA-21 and miRNA-27b, have been implicated in the pathogenesis and progression of various diseases, including OLP. Their altered expression in saliva may provide diagnostic and prognostic insights for this condition. This systematic review examines the expression profiles of miRNA-21 and miRNA-27b in the saliva of OLP patients to assess their potential as biomarkers. The review was conducted in accordance with PRISMA guidelines and was registered in the PROSPERO database. A comprehensive search was conducted in PubMed, Embase, and Scopus using specific keywords. Retrieved titles and abstracts were screened based on predefined eligibility criteria, and relevant studies were analyzed. The initial search identified 71 studies. After screening, 17 abstracts were selected for full-text review. Following evaluation, 11 studies were excluded, resulting in 6 studies being included. Findings indicate a consistent upregulation of miRNA-21 and a downregulation of miRNA-27b in OLP saliva samples. These alterations suggest a potential role in disease pathogenesis and risk assessment. The dysregulation of miRNA-21 and miRNA-27b in OLP underscores their potential as salivary biomarkers for diagnosis and disease monitoring. Moreover, the non-invasive nature of salivary miRNAs offers promising clinical applications, enhancing early detection and personalized management strategies for OLP.

## 1. Introduction

### 1.1. Oral Lichen Planus

Oral lichen planus (OLP) is a chronic autoimmune mucocutaneous disease characterized by T-cell-mediated inflammation and epithelial destruction. Affecting approximately 1–2% of the global population, OLP presents clinically as reticular, atrophic, erosive, or plaque-like lesions. The lesions can cause significant discomfort, including pain, burning sensations, and difficulties in eating and speaking, thereby affecting patients’ quality of life [1]. Furthermore, OLP is classified as a potentially malignant disorder by the World Health Organization, with an estimated malignant transformation rate of 1–1.4% [2,3,4,5]. The pathogenesis of OLP remains incompletely understood, but it is believed to involve a complex interplay of genetic, environmental, and immunological factors. Central to its pathology is the dysregulation of immune responses, particularly involving T cells and cytokine networks, which leads to chronic inflammation and epithelial apoptosis [6]. Recent research has highlighted the role of microRNAs (miRNAs) as key regulators of the abovementioned processes, offering new insights into the molecular mechanisms underlying OLP [7,8,9].

### 1.2. MicroRNAs

MicroRNAs are small, non-coding RNA molecules that regulate gene expression post-transcriptionally, influencing various biological processes, including cell proliferation, differentiation, and apoptosis [10]. Aberrant expression of miRNAs has been implicated in numerous diseases, including inflammatory [11,12] and autoimmune conditions [13] as well as cancers [14]. In the context of OLP, miRNA-21 and miRNA-27b have emerged as particularly significant [15,16]. miRNA-21 is one of the most extensively studied miRNAs due to its role as an oncogene in various cancers and its involvement in promoting inflammation and fibrosis [17]. Studies have shown that miRNA-21 is upregulated in OLP tissues, particularly in erosive and atrophic subtypes, suggesting a link with disease severity and progression. Its overexpression is associated with the activation of pathways such as NF-κB and STAT3, which contribute to sustained inflammation and resistance to apoptosis. Additionally, miRNA-21 has been implicated in altering the tumor microenvironment, fostering conditions conducive to malignant transformation in chronic inflammatory settings like OLP [18,19]. The miRNA-27 family comprises miR-27a and miR-27b, which are transcribed from different chromosomes and differ in their nucleotide sequence at the 3′ end [20]. In vivo and in vitro studies have demonstrated that miR-27b promotes angiogenesis, while its inhibition disrupts embryonic vessel formation, impairs endothelial cell sprouting, and reduces the number of perfused vessels. However, the precise role of miR-27b in endothelial cells remains largely unclear [21]. Consequently, whether miR-27 modulation affects key pathways involved in endothelial homeostasis and represents a potential therapeutic target for improving endothelial function has not been fully investigated [11]. Conversely, miRNA-27b often acts as a tumor suppressor and is involved in immune regulation and epithelial homeostasis [22]. In OLP, miRNA-27b exhibits consistent downregulation, with its levels decreasing further in more severe clinical subtypes. This reduction may diminish its capacity to regulate pro-inflammatory and pro-apoptotic signals, thereby contributing to disease progression. Additionally, the variation in miRNA-27b expression between reticular and erosive OLP suggests its potential utility as a biomarker for disease activity. Recent studies indicate that miRNA-27b plays a role in key pathways involved in epithelial integrity, reinforcing its significance in OLP pathogenesis [23]. The role of salivary miRNAs as non-invasive biomarkers has also gained significant attention [24]. Saliva, being in direct contact with oral lesions, offers a unique medium for detecting molecular changes associated with OLP. Studies have demonstrated that miRNA-21 and miRNA-27b can be reliably quantified in saliva, supporting their use in early diagnosis and monitoring of disease progression. Furthermore, the stability of miRNAs in saliva and their protection within extracellular vesicles enhance their clinical utility. Salivary miRNA profiling represents a promising avenue for personalized medicine, allowing clinicians to monitor disease activity and response to treatment with minimal patient discomfort.

### 1.3. Aim

This review systematically examines the expression patterns of miRNA-21 and miRNA-27b in the saliva of OLP patients, integrating findings from recent studies to evaluate their potential as biomarkers for diagnosis, prognosis, and therapeutic targeting. By addressing gaps in the current understanding and highlighting the clinical implications of miRNA dysregulation, this review aims to contribute to the development of personalized approaches for managing OLP. It also explores the broader implications of miRNA research in enhancing our understanding of autoimmune and inflammatory diseases.

## 2. Materials and Methods

### 2.1. Study Design and Registration

This systematic review was conducted in accordance with PRISMA guidelines and registered in the PROSPERO database (Registration ID: CRD42024613801).

### 2.2. Search Strategy

A comprehensive literature search was performed in PubMed, Embase, and Scopus using the following MeSH terms:

Query 1: (“mirna 21” OR “microRNA 21” OR “mirna 27b” OR “microRNA 27b”) AND (“oral lichen planus” OR OLP OR lichen)

Query 2: (“mirna 21” OR “microRNA 21” OR “mirna 27b” OR “microRNA 27b”) AND (“oral lichen planus” OR OLP OR lichen) AND Saliva.

Studies published until May 2025 were included. Duplicates were removed, and titles, abstracts, and full texts were screened for eligibility. The selection criteria used to include the studies were as follows: (i) studies analyzing miRNA-21 and/or miRNA-27b expression in saliva of OLP patients; (ii) English-language publications. Reviews and meta-analyses were excluded. A structured approach was used to formulate the research question using five components (“PICOS”) [25]. The study population (P) consisted of women and men with oral lichen planus; the intervention (I) was the dosage, using all the possible microbiological analyses, of the abovementioned miRNA in saliva of patients with OLP; the comparison (C) was the assessment of the abovementioned miRNA in saliva of patients without OLP; the outcome (O) was the presence/absence of the abovementioned miRNA in saliva of patients with or without OLP; and the study design (S) included cross-sectional studies, retrospective cohort studies, prospective comparative studies, case–control studies, case series, and case reports. Three independent reviewers (A.R., F.F., and A.L.) extracted data on study characteristics, patient demographics, sample types, miRNA detection methods, and key findings. Discrepancies were resolved through discussion. Data synthesis focused on identifying patterns of miRNA expression and their correlations with clinical and histopathological features of OLP. Quality assessment of non-randomized studies was assessed for all included studies using the Risk of Bias in Non-randomized Studies of Interventions (ROBINS I) [26]. The domains evaluated included bias due to confounding, selection of participants, classification of interventions, deviations from intended interventions, missing data, measurement of outcomes, and selection of reported results.

## 3. Results

**Figure 1:**
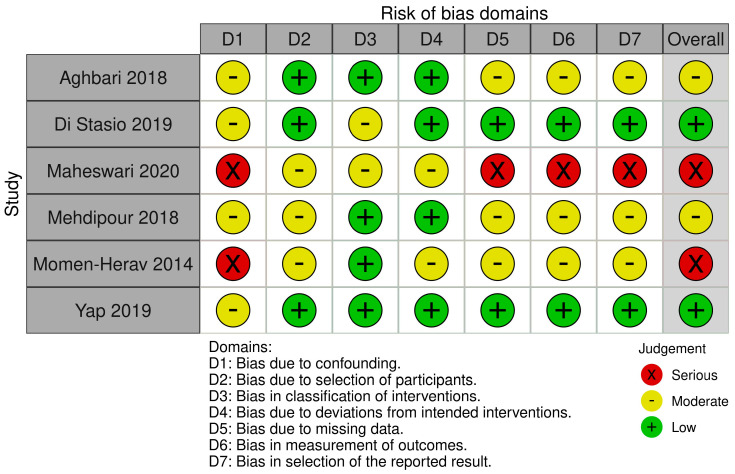
Traffic lights plot for ROBINS analysis [15,19,24,27,28,29].

The results of ROBINS analysis summarized using the robvis tool are provided in Figure 1.

The paper by Yap 2019 and Di Stasio 2019 provided the most reliable evidence [15,19]. Maheswari 2020 and Momen-Herav 2014 should be interpreted cautiously due to high risk of bias [24,28]. Overall, the risk of bias domain showed two studies (Maheswari 2020 & Momen-Herav 2014 [24,28]) had serious risk of bias, primarily due to confounding, missing data, and measurement issues, while four studies (Aghbari 2018, Di Stasio 2019, Mehdipour 2018, Yap 2019 [15,19,27,29]) had moderate to low risk, making them more reliable.

In Aghbari et al. (2018) [27], the overall risk of bias was considered moderate. The study did not adjust for key confounding factors such as smoking and inflammation, which may influence miRNA expression levels. However, participant selection was clearly defined, and exposure classification was strong, with RT-qPCR used with appropriate controls. Missing data were not addressed, and the outcome measurement lacked precise threshold definitions. While diagnostic metrics were reported, the emphasis was on significant findings, with limited discussion of non-significant results. Di Stasio et al. (2019) [15] demonstrated a low overall risk of bias. The study included clearly defined OLP patients and used validated methods for measuring miRNA expression. Although potential confounders were not adjusted for, the study had strong reporting practices and complete datasets. Although diagnostic accuracy measures such as sensitivity and specificity were not calculated, the fold changes and statistical significance were well reported. Maheswari et al. (2020) [28] presented a serious risk of bias. It did not adjust for confounding variables and had vague participant inclusion criteria. Although qRT-PCR was used, the lack of normalization detail, limited validation, and small sample size reduced confidence in the findings. The study also lacked clarity on how missing data were handled. Despite reporting an ROC analysis for miRNA-21, methodological details were sparse, and the results focused exclusively on statistically significant findings. Mehdipour et al. (2018) [19] had a moderate overall risk of bias. While patients were well characterized and exposure classification was appropriate, the study did not control for potential confounders such as demographic variables. Missing data handling was not described. Results for miRNA-21 and miRNA-125a were emphasized, but the presentation of less significant miRNA findings was limited. Diagnostic potential was discussed, though ROC analyses were not performed. In Momen-Herav et al. (2014) [24], the risk of bias was serious. The study’s primary focus was on OSCC, and OLP-specific analyses were limited. Confounding factors were not adjusted for, and participant stratification was unclear. Although reliable methods were used to classify exposure, diagnostic metrics were presented for OSCC only. Reporting was aggregate, with insufficient emphasis on OLP-related results. Yap et al. (2019) [29] was assessed as having a low risk of bias overall. The study employed validated detection methods and included a large, well-characterized patient cohort. Although confounders were not adjusted for, the study design and execution were robust. However, as the focus was on OSCC risk classification, the diagnostic value of miRNA-21 and miRNA-27b in OLP specifically was not clearly delineated. Still, the reporting was transparent and comprehensive.

**Figure 2:**
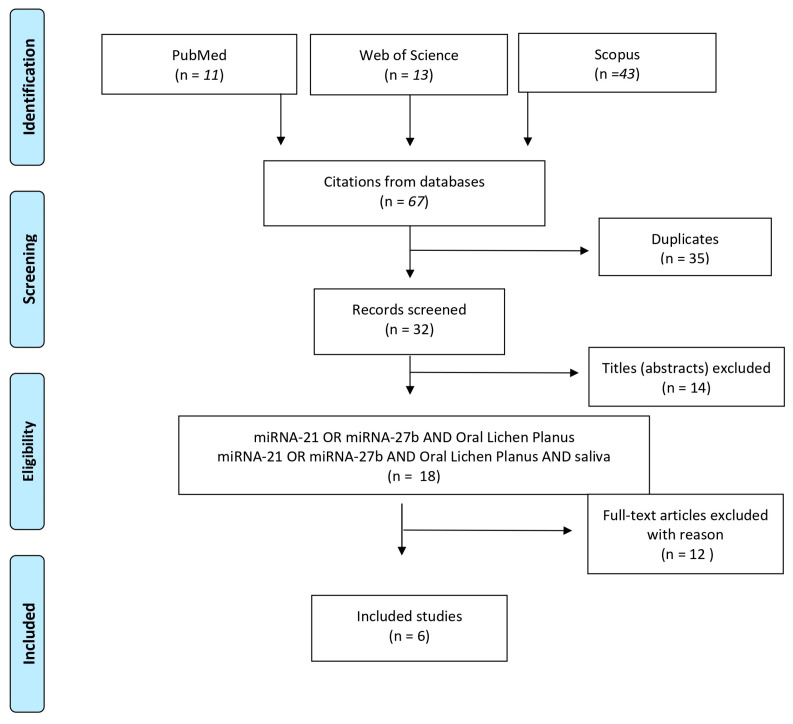
PRISMA flow chart.

The initial electronic search yielded 67 results. Titles and abstracts retrieved from the search were independently screened by three authors (A.R., A.L., and F.F.) based on the predefined criteria. Of the 67 initial results, 35 were identified as duplicates, and 14 were excluded after screening titles according to the exclusion criteria (review articles). Subsequently, 18 abstracts were analyzed, and full-text articles were obtained for all titles agreed upon by the authors. Any disagreements were resolved through discussion. Reading the full texts, 12 other articles were excluded, resulting in 6 studies being included in this review. The flowchart in Figure 2 outlines the steps of the selection process, while Table 1 provides a summary and schematic representation of the included studies.

### 3.1. miRNA-21

Di Stasio et al. conducted a study to identify microRNAs (miRNAs) involved in the pathogenesis of oral lichen planus (OLP) by analyzing their expression levels in saliva samples from OLP patients compared to healthy controls. Using microarray technology, they detected significant differences in 98 miRNAs, with 96 showing increased expression and 12 showing decreased expression in OLP samples (Table 1). Among the upregulated miRNAs, miR-21 exhibited the most significant increase, with levels approximately three times higher in OLP patients compared to healthy controls (*p* < 0.001, Student’s *t*-test) [15]. Maheswari et al. conducted a study to evaluate the expression levels of salivary miRNA-21 and miRNA-31 in patients with oral potentially malignant disorders (OPMD), comparing them to healthy individuals. Their objective was to analyze differences in miRNA expression across various types of OPMD lesions and different grades of dysplasia, ultimately identifying the most sensitive and specific miRNA for detecting early dysplastic changes. For this study, saliva samples were collected from 9 patients with oral lichen planus (OLP) and 36 healthy individuals. The results revealed a significant upregulation of miRNA-21 in OLP patients compared to the control group. In particular, miR-21 levels showed a 2.03-fold increase, with a statistically significant *p*-value of 0.002 [28]. Mehdipour et al. conducted a study to evaluate the diagnostic and prognostic potential of salivary miR-21 in patients with oral lichen planus (OLP)—both with and without dysplasia—as well as in patients with oral squamous cell carcinoma (OSCC). The study included 30 patients diagnosed with OLP, who were further classified based on histopathological findings: 10 patients showed no signs of dysplasia, while 20 patients exhibited dysplastic changes. Additionally, 15 OSCC patients served as a positive control group, selected according to clinical and histopathological criteria. To establish a healthy baseline, the study also included 15 carefully matched healthy individuals as a negative control group, ensuring similarity in age and gender. When analyzing miR-21 expression across the different groups, the researchers found that miR-21 levels were significantly elevated in OSCC patients compared to those with OLP, regardless of dysplasia status. The difference was highly statistically significant, with *p*-values of <0.0001, 0.0017, and 0.012 when comparing OSCC to OLP with dysplasia, OLP without dysplasia, and healthy controls, respectively. The lowest miR-21 levels were observed in healthy individuals. Moreover, the authors observed a notable elevation in miR-21 levels in saliva samples from dysplastic OLP patients compared to those without dysplasia. However, a direct comparison between OLP patients and healthy controls was not performed in this study [19]. Yap et al. explored the effectiveness of a previously developed test in evaluating the risk of oral squamous cell carcinoma (OSCC) in patients with oral lesions using an algorithm-based classification system to analyze microRNA expression in oral swirls. Their study aimed to determine whether this approach could accurately differentiate between individuals with and without mucosal abnormalities, offering a predictive tool for assessing OSCC risk in oral potentially malignant disorders (OPMDs) [29]. In their 2018 research, Yap and colleagues identified a panel of OSCC-associated microRNAs, including miR-24-3p, miR-21-5p, let-7c-5p, miR-99a-5p, and miR-100-5p, which were used to develop a scoring system known as the dysregulation score (dSCORE). By integrating this score with a predictive algorithm, they were able to detect the presence of OSCC with high accuracy based on the abundance of these microRNAs in oral swirls [30]. Their study included two main groups: individuals with visible mucosal abnormalities (MA) and those without mucosal abnormalities (NMA). Among those with mucosal abnormalities, some had histologically normal epithelium (HNE), such as fibroepithelial polyps or denture-associated hyperplasia, while others were diagnosed with OPMD or OSCC. The OPMD group encompassed a variety of conditions, including oral lichen planus (OLP), oral lichenoid lesions, leukoplakia (with and without dysplasia), and traumatic ulcerations with stromal eosinophils. Despite the study’s valuable contributions to OSCC risk assessment, a key limitation is that the authors did not specifically isolate data related to OLP. Instead, they classified cases broadly under OPMD and OSCC, without analyzing miRNA-21 expression in OLP patients separately. Consequently, while their findings provide insights into the potential of microRNA-based diagnostics for OSCC, they do not offer specific guidance for diagnosing or monitoring OLP, making the study less relevant for this particular condition [29].

### 3.2. miRNA-27b

Aghbari et al. conducted a study to investigate the expression of microRNA-27b and microRNA-137 in both tissue and saliva samples from patients with OLP and healthy individuals. The primary goal was to determine whether these microRNAs could serve as biomarkers for monitoring disease activity and assessing the risk of malignant transformation.

The study involved 20 patients diagnosed with OLP and 20 healthy individuals as a control group. All patients in the OLP group were free from other systemic or oral diseases and had not received any treatment for at least three months prior to biopsy collection. To further explore the variations within the disease, the OLP group was categorized into three subtypes based on clinical presentation: (a) papular, reticular, or plaque type; (b) atrophic type; and (c) erosive type.

One of the key aspects of the study was comparing miRNA-27b expression in saliva between OLP patients and healthy controls. The results revealed that miRNA-27b levels were significantly lower in OLP patients, with a mean value of 5.343, compared to 10.592 in the control group. However, when comparing the different OLP subtypes among themselves, no statistically significant differences in miRNA-27b expression were found.

Interestingly, when the OLP subgroups were compared with the control group, subgroups (a) and (b) exhibited significantly lower miRNA-27b expression levels (with *p*-values of 0.001 and 0.048, respectively). However, subgroup (c) showed no significant difference in miRNA-27b levels compared to the control group, suggesting a potential variation in disease behavior among the subtypes.

To establish a potential diagnostic threshold, the study identified a cutoff value of 8.04 for miRNA-27b expression in saliva. Individuals with expression levels below this value were classified as having OLP, whereas those with levels above 8.04 were considered healthy.

In terms of diagnostic accuracy, salivary miRNA-27b demonstrated a fair level of reliability, with an AUC (area under the curve) of 0.78, a sensitivity of 75%, and a specificity of 100% at the determined cutoff value. These findings suggest that miR-NA-27b in saliva could be a promising, non-invasive biomarker for diagnosing OLP and potentially monitoring disease progression [27].

Di Stasio et al. also identified a notable downregulation of miR-27b in OLP saliva samples. The microarray analysis revealed that miR-27b levels were reduced by approximately threefold in OLP patients compared to normal controls (*p* < 0.001, Student’s *t*-test). This finding was further validated using qRT-PCR, confirming the consistent downregulation of miR-27b in the saliva of all OLP patients examined [15]. Momen-Heravi et al. investigated the variations in microRNA expression among four distinct groups: patients diagnosed with OSCC, individuals in remission from OSCC (OSCC-R), patients with OLP, and healthy controls (HCs). Their findings revealed that miRNA-27b levels were notably elevated in OSCC patients compared to healthy individuals, OSCC-R patients, and those with OLP. This significant increase in expression suggests that miRNA-27b may serve as a key biomarker for OSCC. Additionally, receiver operating characteristic (ROC) curve analysis confirmed that miRNA-27b holds strong potential as a diagnostic tool, effectively differentiating OSCC patients from those in the other groups. However, a direct comparison between OLP patients and healthy controls was not performed [24]. The results of the main results highlighted are summarized in Table 2.

## 4. Discussion

This study underscores the consistent upregulation of miRNA-21 and the downregulation of miRNA-27b in the saliva of OLP patients. The upregulation of miRNA-21 aligns with its role in promoting inflammatory and oncogenic pathways, reinforcing its potential as a biomarker for high-risk OLP subtypes. Studies have reported elevated miRNA-21 expression in both saliva and tissue samples of OLP patients, further emphasizing its diagnostic utility. Danielsson et al. (2011) demonstrated significant miRNA-21 overexpression in erosive and atrophic OLP subtypes, correlating with reduced p53 expression. Given p53’s crucial role in tumor suppression, its downregulation in conjunction with miRNA-21 upregulation suggests a potential mechanism linking chronic inflammation to neoplastic transformation in OLP [31]. Similarly, Mehdipour et al. (2018) highlighted the diagnostic potential of miRNA-21, showing its significantly elevated levels in dysplastic OLP compared to non-dysplastic subtypes and controls (*p* = 0.0017). This suggests that miRNA-21 expression may help distinguish between benign and potentially malignant OLP cases, making it a candidate for risk stratification in clinical settings [19]. Furthermore, Madkour et al. (2012) reported a 3.2-fold increase in miRNA-21 levels, linking its upregulation to NF-κB pathway activation, a well-established mediator of chronic inflammation and immune response dysregulation. The ability of miRNA-21 to interact with pro-inflammatory signaling pathways supports its role in exacerbating the inflammatory microenvironment of OLP, potentially accelerating disease progression and malignant transformation. The integration of miRNA-21 expression profiling into non-invasive saliva-based diagnostic tests could improve early detection and disease monitoring while minimizing the need for invasive biopsies [32]. In contrast to miRNA-21, miRNA-27b exhibits a consistent downregulation in OLP, suggesting its involvement in immune regulation and epithelial homeostasis. As a member of the miR-232724 cluster, miRNA-27b plays a role in modulating inflammatory responses, keratinocyte differentiation, and apoptosis, making its dysregulation particularly relevant in OLP pathogenesis. Several studies confirm the significant downregulation of miRNA-27b in both tissue and saliva samples of OLP patients, reinforcing its role in disease progression. Aghbari (2018) observed reduced miRNA-27b expression across all OLP subtypes, with statistical differences between reticular and erosive forms, indicating a possible link between miRNA-27b levels and disease severity [27]. Similarly, Zhang et al. (2012) confirmed a 5.5-fold reduction in miRNA-27b expression in atrophic–erosive OLP compared to controls, correlating with higher disease activity and severity. This suggests that miRNA-27b may act as a tumor suppressor, and its downregulation could contribute to increased epithelial cell proliferation, impaired apoptosis, and persistent inflammation [23]. Further mechanistic insights into miRNA-27b function were provided by Chen et al. (2019), who explored its interaction with cyclophilin D and PLK2—two proteins involved in keratinocyte apoptosis and proliferation. Their findings highlight how miRNA-27b may regulate epithelial turnover in OLP, and its suppression could disrupt epithelial barrier integrity, leading to persistent lesions and increased susceptibility to malignant changes [33]. These results align with broader miRNA research in chronic inflammatory and autoimmune diseases, where miRNA-27b downregulation has been implicated in dysregulated immune signaling and epithelial dysfunction. The accumulating evidence from multiple studies highlights the potential role of salivary miRNA profiling in the early detection of OLP. Among the studied miRNAs, miRNA-21’s levels appear to be particularly relevant for assessing disease severity and estimating the risk of malignant transformation. As saliva-based diagnostics offer a non-invasive alternative to tissue biopsies, miRNA profiling could provide a practical and efficient tool for clinicians to monitor OLP progression and treatment response. Polizzi et al. (2023) further expanded on the miRNA landscape in OLP by identifying differential expression of additional miRNAs, such as miRNA-125b and miRNA-203, emphasizing their roles in disease severity and potential malignant transformation. This suggests that a panel of miRNAs, rather than a single biomarker, may enhance diagnostic accuracy [34]. Similarly, Di Stasio et al. (2019) validated the utility of salivary miRNA-27b as a non-invasive biomarker for monitoring OLP progression, reinforcing the potential clinical relevance of salivary miRNA detection [15]. Moreover, Prasad et al. (2017) developed an miRNA panel specifically designed to differentiate OLP from other potentially malignant disorders, underscoring the specificity of miRNA-21 and miRNA-27b in OLP. Their work suggests that miRNA-based biomarkers could be integrated into routine clinical workflows, potentially allowing for early risk assessment, timely interventions, and personalized treatment approaches [35]. While the findings of this systematic review strongly support the clinical relevance of miRNA-21 and miRNA-27b in OLP, several limitations must be acknowledged:

Heterogeneity of study methodologies: Differences in sample collection methods, normalization techniques, and detection platforms introduce variability in reported findings. One of the main issues highlighted by our study is the heterogeneity in diagnostic criteria for OLP across the literature. For example, in the study by Mehdipour et al. [19], the authors analyzed a cohort of 30 patients diagnosed with OLP, 20 of whom presented with dysplasia. However, according to the World Health Organization (WHO) diagnostic criteria, the presence of epithelial dysplasia excludes a definitive diagnosis of OLP. Therefore, these cases do not strictly fulfill the criteria for OLP, raising concerns about case classification and study comparability.

Small sample sizes: Many included studies had limited patient cohorts, which may affect the generalizability of results.

Lack of longitudinal data: Most studies provided cross-sectional analyses, making it difficult to establish causal relationships between miRNA expression and disease progression.

Limited analysis of salivary miRNA-27b: While miRNA-21 has been extensively validated, data on salivary miRNA-27b expression remain scarce.

## 5. Conclusions

Overall, this study provides compelling evidence supporting the diagnostic and prognostic potential value of miRNA-21 and miRNA-27b in OLP. Their differential expression profiles offer valuable insights into disease mechanisms, risk stratification, and potential treatment targets. Future research should prioritize standardizing miRNA detection methods, conducting large-scale multicenter studies, and incorporating longitudinal analyses to track miRNA expression changes over time. As miRNA research advances, integrating miRNA biomarkers into clinical practice could revolutionize early detection, personalized management, and therapeutic innovation in OLP and other related conditions.

## Figures and Tables

**Table 1:** Characteristics of the six included studies.

Authors	Aim	Sequencing Method	Patients	OLP Pattern	miRNA Investigated	Main Results
Aghbari, 2018 [27]	To compare the expression of miRNA-27b and miRNA-137 in tissues and saliva between OLP patients and controls.	qRT-PCR	20 OLP + 20 controls	5 white, 7 erosive, 8 atrophic	27b, 137	miRNA-27b and miRNA-137 were downregulated in patients affected by OLP. A statistical difference was found between OLP and control groups in both saliva and tissue samples, with no statistical difference between each OLP subgroup for miRNA27b. A statistical difference was also found between the saliva samples of each OLP subgroup.
Di Stasio, 2019 [15]	To analyze the expression of salivary miR-27b in oral lichen planus (OLP) patients using microarray technology and qRT-PCR validation and explore its potential as a non-invasive biomarker for OLP diagnosis and disease activity monitoring.	qRT-PCR, microarray analysis	5 OLP + 5 controls	3 atrophic/erosive, 2 mixed	15b, 21, 27b, 125b, 203	miR-27b was downregulated in the saliva of all OLP patients (~3-fold decrease compared to controls, *p* < 0.001), while miR-15b, miR-21, miR-125b, and miR-203 were upregulated (miR-15b by ~85-fold). Validation confirmed the downregulation of miR-27b, suggesting its role in keratinocyte activity and OLP pathogenesis. Salivary collection was a well-tolerated, non-invasive procedure, highlighting its potential for clinical applications.
Maheswari, 2020 [28]	To evaluate the expression levels of salivary miRNA-21 and miRNA-31 in oral potentially malignant disorders (OPMD) and assess their diagnostic potential as biomarkers for early malignant changes.	qRT-PCR	12 OSF, 8 OL, 9 OLP, 7 OSF + OL + 36 controls	n.s.	21, 31	miR 21 was significantly upregulated in OPMD patients compared to controls (2.44-fold in leukoplakia and 2.03-fold in oral lichen planus, *p* < 0.05). It was associated with severe dysplasia (3.6-fold increase, *p* < 0.01). Area under the curve (AUC) was 0.82, with 69% sensitivity and 66% specificity. miR-31 was elevated in OPMD (1.6-fold in leukoplakia and 1.2-fold in oral lichen planus, *p* > 0.05) and significantly associated with severe dysplasia (2.5-fold increase, *p* < 0.01). AUC was 0.51, with lower diagnostic efficiency compared to miRNA-21. Salivary miRNA-21 demonstrated potential as a non-invasive biomarker for detecting early malignant changes in OPMD.
Mehdipour, 2018 [19]	To evaluate the diagnostic and prognostic potential of salivary miRNAs in OLP patients with and without dysplasia and their relationship to malignant transformation.	qRT-PCR	30 OLP (20 dysplasia), 15 OSCC + 15 controls	n.s.	21, 125a, 31, 200a	miR-21 was significantly increased in OLP (*p* = 0.012), dysplastic OLP (*p* = 0.0017), and OSCC patients (*p* < 0.0001) compared to controls. miR-125 was significantly decreased in OLP (*p* < 0.001), dysplastic OLP (*p* < 0.0001), and OSCC (*p* < 0.0001), with lower levels in dysplastic OLP compared to non-dysplastic OLP (*p* = 0.002). miR-31 was elevated in dysplastic OLP (*p* = 0.01) and OSCC (*p* = 0.004) but not significantly altered in non-dysplastic OLP. miR-200a showed no significant changes in OLP patients with or without dysplasia but was significantly reduced in OSCC patients (*p* < 0.0001). Increased miR-21 and decreased miR-125a levels may indicate poor prognosis in OLP, while lack of significant changes in miR-31 and miR-200a may suggest absence of malignant transformation.
Momen-Herav, 2014 [24]	To evaluate the differential expression of miRNAs in the saliva of patients with oral squamous cell carcinoma (OSCC), patients in remission from OSCC (OSCC-R), patients with oral lichen planus (OLP), and healthy controls to identify potential salivary biomarkers for OSCC diagnosis.	NanoString nCounter miRNA expression assay, with validation by real-time quantitative polymerase chain reaction (RT-qPCR)	34 patients: 9 with OSCC, 8 with OSCC in remission (OSCC-R), 8 with OLP, and 9 healthy controls (HCs)	n.s.	Over 700 miRNAs were analyzed, and 13 miRNAs were found to be significantly deregulated in OSCC patients compared to healthy controls, as follows: Downregulated: miRNA-136, miRNA-147, miRNA-1250, miRNA-148a, miRNA-632, miRNA-646, miRNA-668, miRNA-877, miRNA-503, miRNA-220a, and miRNA-323-5p. Upregulated: miRNA-24, miRNA-27b	miRNA-27b was found to be significantly upregulated in OSCC patients compared to those with OSCC-R, OLP, and healthy controls, suggesting its potential as a diagnostic biomarker for OSCC. miRNA-136 was found to be downregulated in OSCC patients compared to healthy controls and OSCC-R patients, making it useful for distinguishing OSCC from OSCC-R. ROC curve analysis showed that miRNA-27b and miRNA-136 had high sensitivity and specificity in distinguishing OSCC patients from other groups.
Yap 2019 [29]	To evaluate a panel of OSCC-associated microRNAs in oral swirls as a non-invasive diagnostic tool for OSCC and OPMDs, including OLP, using dSCORE and an algorithm for risk classification.	qRT-PCR	53 OSCC, 31 OLP, 15 OLL, 26 OL, 2 TUGSE + 63 controls	n.s.	24-3p, 21-5p, 99a-5p, 100-5p	dSCORE and the algorithm demonstrated 86.8% sensitivity, 81.5% specificity, and 84.2% accuracy for OSCC diagnosis. miR-24-3p was deregulated in OSCC and was linked to cell proliferation, cell cycle, and apoptosis. miR-21-5p was upregulated in OSCC and was strongly associated with oncogenic processes. miR-99a-5p and miR-100-5p both targeted tumor-related pathways (e.g., mTOR and PI3K) and contributed to differentiating OSCC from OPMDs. The identified miRNA panel effectively distinguished OSCC from OPMDs and highlighted the potential of miRNA-based tools for non-invasive monitoring and early detection of malignant transformations in disorders like OLP.

n.s. = not specified.

**Table 2:** Expression of miRNAs 27b and 21 in patients with oral lichen planus.

miRNA.	Aghbari, 2018 [27]	Di Stasio, 2019 [15]	Maheswari, 2020 [28]	Mehdipour, 2018 [19]	Momen-Heravi, 2014 [24]	Yap, 2019 [29]
miR-27b	downregulated	downregulated	-	-	upregulated	-
miR-21	-	upregulated	upregulated	upregulated	-	upregulated

## Abbreviations

Oral Lichen Planus	OLP
Area Under the Curve	AUC
Quantitative Reverse Transcription Polymerase Chain Reaction	qRT-PCR
Oral Potentially Malignant Disorders	OPMDs
Oral Squamous Cell Carcinoma	OSCC
